# The effect of subsequent immunosuppressant use in organ-transplanted patients on prostate cancer incidence: a retrospective analysis using the Korean National Health Insurance Database

**DOI:** 10.1186/s12894-021-00883-8

**Published:** 2021-08-21

**Authors:** Hyung Ho Lee, Jae Young Joung, Sung Han Kim

**Affiliations:** Department of Urology, Urologic Cancer Center, Research Institute and Hospital of National Cancer Center, 323 Ilsanro Ilsandonggu Madoodong, Goyang, 10408 Republic of Korea

**Keywords:** Prostate cancer, Incidence, Overall survival, Organ transplantation risk factor

## Abstract

**Backgrounds:**

Prostate cancer (PC) is the most common solid organ cancer. However, there is still no definite consensus before and after organ transplantation (TPL). We aimed to analyze whether PC incidence increased in TPL patients with subsequent use of immunosuppressants using the Korean National Health Insurance Database.

**Methods:**

TPL patients between 2003 and 2015(N = 12,970) were age- and year-matched to non-TPL patients (N = 38,910) in a 1:3 ratio. Multivariate Cox regression analysis adjusted for significant prognostic clinicopathological parameters, including the duration of immunosuppressant agent use (0–300 or > 300 days), and Kaplan–Meier analysis with log-rank test were used to evaluate the association of TPL with PC incidence between the groups.

**Results:**

Median overall survival was 4.86 years; overall mortality rate was 3.4% (n = 1761). Regardless of differences in baseline characteristics between the groups, multivariate analysis for PC incidence showed that age, immunosuppressant use, and TPL organ subtypes were significant factors for the overall population, whereas only age was significant in the TPL group (*p* < 0.05). After adjusting for age, underlying disease, and prescribed medication (aspirin, statin), multiple subgroup analysis models for PC incidence were evaluated. PC incidence was increased in the TPL group (hazard ratio [HR] 1.965, *p* < 0.001); however, PC incidence in the TPL group became insignificant after adjusting for immunosuppressant use (*p* = 0.194). Kaplan–Meier curves also showed that PC incidence was significantly different according to age and TPL with the use of immunosuppressants between the TPL and non-TPL groups.

**Conclusions:**

PC incidence was higher in the TPL group using immunosuppressants than in the non-TPL group.

*Trial registration*: The study was retrospectively registered.

**Supplementary Information:**

The online version contains supplementary material available at 10.1186/s12894-021-00883-8.

## Background

Organ transplantation (TPL) has been markedly successful in the last several decades, with an approximately 50% increase in the survival rate over 50 years after surgery, even though the incidence of end-stage organ failure has also increased [1, 2]. However, the estimated prevalence of cancer has been found to be 2.5–3.5 times higher in TPL patients during long-term immunosuppressive therapy due to the reduced immune surveillance of neoplastic cells and the development of post-TPL opportunistic infections [3, 4]. Post-TPL malignancies have poor prognosis and biologically aggressive features, resulting in a tenfold higher mortality than that of the normal cancer population [5]. This is because post-TPL patients may receive less aggressive cancer treatment, have higher comorbidities, and are at a risk of TPL rejection. Oncogenesis and the pathophysiology of cancer progression during immunosuppression in TPL patients remain unclear; thus, establishing screening and treatment guidelines for post-TPL malignancies is extremely challenging.

The worldwide prostate cancer (PC) incidence increases rapidly and proportionally in men with longer lifespans. PC is the most common solid organ cancer in males and the third most common cause of cancer mortality in the general population [6]. Given that TPL increases the recipients’ lifespan to approximately that of the normal population, the risk of PC also increases in surviving TPL patients. However, there is no consensus on post-TPL PC screening and management, and there are no randomized studies on this subject, except for several prospective and retrospective studies [7]. Therefore, in this study, we used a large population-based National Health Insurance Claim dataset and investigated the effect of subsequent immunosuppressant use in TPL patients according to its duration in TPL patients and non-TPL patients.

## Methods

### Study design and data source

This retrospective, population-based study used data from the Korean National Health Insurance database managed by the NHIS. The NHIS database provides comprehensive health insurance coverage for all citizens in Korea. The population-based Korean cohort registry included information on the International Classification of Diseases, Tenth Revision, Clinical Modification (ICD-10-CM) codes.

In this study, the definitions of all the measurements were also retrieved using the ICD-10-CM codes. The ICD-10 codes for PC (C61) and TPL (V42.0-9) were used to obtain and analyze the data of 5,580,495 Korean men under NHI coverage. Patient privacy was ensured by anonymizing identifiers and other personal information from the NHIS. Preoperative underlying diseases were also searched using the ICD-10-CM codes for hypertension (HTN, I10-13, I15), diabetes mellitus (DM, E11-14), and dyslipidemia (E78). We included only TPL of the kidney (V42.0), liver (V42.7), lung (V42.6), pancreas (V42.83), and heart (V42.1). Post-TPL PC was defined as the first occurrence of PC without a prior existence of the cancer according to the recorded ICD-9-CM C61 code. OS was estimated until the end of 2018, with death defined as the matched death code or termination from the NHI program.

Demographic characteristics, such as age, medication (aspirin, statin), and comorbidities (hypertension, diabetes, and dyslipidemia), and the duration of immunosuppressant use (≤ or > 300 days) were analyzed as risk factors for post-TPL PC. The cut-off point of 300 days was decided after the distribution analysis (< 3, 3–6, 6–9, 10–12, and > 12 months) from the dataset indicated that a 10-month cut-off showed significant differences between groups and almost all subjects in the TPL group used the immunosuppressant more than 300 days (N = 12,955, 99.88%). All immunosuppressants such as calcineurin inhibitors, corticosteroids, cytotoxic immunosuppressants, immunosuppressant antibodies, sirolimus derivatives, and others such as mycophenolate were included. Among the non-TPL group, corticosteroid use > 300 days was observed in 22.16%.

### Patients

The inclusion criteria were male sex; TPL of either the lung, liver, kidney, or heart; age > 20 years; and availability of health screening data from 2003 to 2015 with a follow-up until the end of 2018. Of the 23,787 male TPL patients identified during the study period (2003–2015), we excluded 10,817 patients because of female sex (N = 8,443), age < 20 years (N = 531), previous history of prostate cancer before TPL (N = 245), follow-up < 1 year (N = 1,233), and other missing variables (N = 365). Other exclusion criteria were intraoperative or postoperative death within 1 month, multiple organ TPL, previous history of any cancers before organ TPL, and any history of skin and corneal TPL surgery (V42.3 and V42.5). The remaining 12,970 patients were matched for age and year of TPL surgery in a 1:3 ratio. Thus, 51,880 patients were included in the analysis. Age was categorized as follows: ≥ 20–49, ≥ 50–64, > 65–74, and ≥ 75 years; while year of TPL was categorized as 2003–2007, 2008–2012, or 2013–2015 (Table 1).

**Table 1:** Baseline demographics of overall prostate cancer patients and comparison between TPL and non-TPL groups

Parameter	Total TPL (N = 51,880)	Non-TPL group (N = 38,910)	TPL group (N = 12,970)	*p* value
	N (%)	N (%)	N (%)	
Age (years; mean, STD)	48.10 (10.24)	48.1 ± 10.24	48.1 ± 10.24	1.000
Age, 20 ≤ Age < 50	25,856 (49.84)	19,392 (49.84)	6464 (49.84)	1.000
50 ≤ Age < 65	24,308 (93.41)	18,231 (93.41)	6077 (93.41)	
65 ≤ Age ≤ 75	1,688 (6.49)	1266 (6.49)	422 (6.49)	
75 < Age	28 (0.11)	21 (0.11)	7 (0.11)	
Diagnostic Year of Prostate Cancer	< .001			
2007–2009	1 (0.53)	0 (0)	1 (1.3)	
2010–2012	40 (21.28)	20 (18.02)	20 (25.97)	
2013–2015	147 (78.19)	91 (81.98)	56 (72.73)	
Year of transplanted surgery	1.000			
2003–2007	12,524 (24.14)	9393 (24.14)	3131 (24.14)	
2008–2012	18,532 (35.72)	13,899 (35.72)	4633 (35.72)	
2013–2015	20,824 (40.14)	15,618 (40.14)	5206 (40.14)	
Underlying disease				
Hypertension	15,511 (29.90)	7376 (18.96)	8135 (62.72)	< .0001
Diabetes	9,504 (18.32)	3253 (8.36)	6251 (48.2)	< .0001
Dyslipidemia	8,546 (16.47)	4461 (11.46)	4085 (31.5)	< .0001
Medication				
Aspirin	1776 (3.42)	803 (2.06)	973 (7.5)	< .0001
Statin	8701 (16.77)	4389 (11.28)	4312 (33.25)	< .0001
Use of Immunosuppressant agents	21,578 (41.59)	8623 (22.16)	12,955 (99.88)	< .0001
Duration of immunosuppressant, ≥ 300 days	11,338 (21.9)	225 (0.6%)	11,113 (85.68)	< 0.001
Transplanted organ				
1. Kidney	7,026 (13.54)	N.A	7026 (54.17)	
2. Liver	5,445 (10.50)	N.A	5445 (41.98)	
3. Pancreas	28 (0.05)	N.A	28 (0.22)	
4. Heart	398 (0.77)	N.A	398 (3.07)	
5. Lung	73 (0.14)	N.A	73 (0.56)	
Charlson Comordity Index Grade 2	< 0.001			
0	23,693 (45.67)	23,689 (60.88)	4 (0.03)	
1	8,231 (15.87)	8126 (20.88)	105 (0.81)	
2 or more	19,956 (38.47)	7095 (18.23)	12,861 (99.16)	
Survival, alive	50,199 (96.61)	38,092 (97.90)	12,027 (92.73)	< 0.001
Survival time (year; mean SD)	4.86 (2.49)	4.9 ± 2.49	4.73 ± 2.49	

*N.A* not available

### Statistical analysis

Data were expressed as mean ± standard deviation and as percentages for continuous and categorical variables, respectively. Multivariate-adjusted Cox regression analysis was performed, and the associated hazard ratio (HR) and 95% confidence interval (CI) for PC prevalence and TPL were analyzed. Calculations were performed after adjusting for age, diabetes, hypertension, dyslipidemia, medication, immunosuppressant drugs, immunosuppressant duration, Charlson Comorbidity Index grade 2, and the year of PC diagnosis and TPL surgery. Kaplan–Meier curves with log-rank tests for PC prevalence and OS were analyzed between the TPL (N = 12,970, 25%) and non-TPL (N = 38,910, 75%) groups. A *p* value of < 0.05 was considered statistically significant.

## Results

The mean patient age was 48.1 (SD: 10.2) years, and the median OS was 4.86 (± 2.49) years. In total, 1761 patients (3.4%) died during the study period (Table 1). The most common transplanted organ was the kidney (13.5%), followed by the liver (10.5%), heart (0.8%), lung (0.1%), and pancreas (0.1%). There were significant differences in baseline characteristics, including the rates of underlying diseases, medication use (statin, aspirin), Charlson Comorbidity Index, duration of immunosuppressant use, year of diagnosis of PC, and survival, between the TPL and non-TPL groups after matching for age and year of TPL surgery (*p* < 0.001, Table 1).

On comparing the TPL and non-TPL groups with the overall population (N = 51,880), the incidence of PC was found to be significantly associated with age (Age: 20–50 years, hazard ratio [HR] 0.113, 95% confidence interval [CI] 0.09–0.186; 65–75 years, HR 2.995, CI 1.948–4.603; > 75 years, HR 13.851, CI 1.918–100.017), duration of immunosuppressant use (> 300 days, HR 3.504, CI 1.345–9.129), and kidney TPL subtype (HR 0.319; *p* < 0.05, Table 2). For the TPL group, multivariate analysis for prevalence of PC age as a significant factor (Age: 20–50 years, HR 0.111, CI 0.05–0.245; 65–75 years, HR 2.809, CI 1.383–5.708) (*p* < 0.001, Table 3). And liver, heart transplantation group were more prevalence tendency of PC compared with kidney transplantation group (Liver; Univariate HR 3.398, CI 2.053–5.626: Heart; Univariate HR 1.850, CI 0.434–87.889: *p* = 0.001, Table 3).

**Table 2:** Univariate and multivariate cox regression for prostate cancer incidence among overall patients

Characteristics	N	Event	Duration	IR (per 1000)	Univariate		Multivariate	
					HR	*p* value	HR	*p* value
Age (years), 20 ≤ Age < 50	25,856	18	135,209.95	0.1331	0.098 (0.06,0.161)	< .0001	0.113 (0.069,0.186)	< .0001
50 ≤ Age < 65	24,308	144	110,258.86	1.306	1 (Ref.)		1 (Ref.)	
65 ≤ Age ≤ 75	1688	25	6550.21	3.8167	3.089 (2.019, 4.726)		2.995 (1.948,4.603)	
75 < Age	28	1	76.54	13.0646	12.92 (1.803,92.597)		13.851 (1.918,100.017)	
Aspirin	1776	13	8017.85	1.62138	2.3 (1.309,4.04)	0.0038	1.295 (0.722,2.324)	0.3863
Statin	8701	37	39,075.1	0.94689	1.364 (0.952,1.955)	0.0905	1.32 (0.774,2.254)	0.3082
Duration of Immunosuppressant agent (> 300 days)	11,338	78	57,837.55	1.3486	2.345 (1.754,3.134)	< 0.001	3.504 (1.345,9.129)	0.0084
Hypertension	15,511	80	72,050.92	1.11033	1.878 (1.407,2.508)	< 0.001	1.347 (0.971,1.87)	0.0746
Diabetes mellitus	9504	64	44,114.7	1.45076	2.461 (1.82,3.328)	< 0.001	1.198 (0.839,1.71)	0.3202
Dyslipidemia	8546	29	37,485.7	0.77363	1.073 (0.722,1.595)	0.7258	0.632 (0.353,1.131)	0.122
Transplanted organ								
Control	38,910	111	190,764.2	0.58187	1 (Ref.)	< .0001	1 (Ref.)	0.0271
1. Kidney	7026	21	33,615.34	0.62471	1.08 (0.677,1.722)		0.319 (0.11,0.922)	
2. Liver	5445	54	25,616.58	2.10801	3.661 (2.645,5.068)		0.838 (0.314,2.24)	
3. Pancreas	28	0	105.38	0	N.A		N.A	
4. Heart	398	2	1760.07	1.13632	2.013 (0.497,8.151)		0.37 (0.068,2.03)	
5. Lung	73	0	233.99	0	N.A		N.A	

*N.A* not available

**Table 3:** Univariate and Multivariate cox regression for prostate cancer incidence only among organ-transplanted patients

Characteristics	N	Event	Duration	IR (per 1000)	Univariate		Multivariate	
					HR	*p* value	HR	*p* value
Age (years), 20 ≤ Age < 50	6464	7	33,223.01	0.2107	0.088 (0.04,0.192)	< 0.001	0.111 (0.05,0.245)	< 0.001
50 ≤ Age < 65	6077	61	26,552.55	2.29733	1 (Ref.)		1 (Ref.)	
65 ≤ Age ≤ 75	422	9	1537.49	5.85368	2.7 (1.339,5.444)		2.809 (1.383,5.708)	
75 < Age	7	0	18.31	0	0 (0)		0 (0)	
Aspirin Yes	973	7	4371.79	1.60118	1.318 (0.606,2.866)	0.486	1.249 (0.568,2.749)	0.5798
Statin Yes	4312	15	19,924.96	0.75282	0.502 (0.286,0.883)	0.017	1.088 (0.513,2.306)	0.8257
Duration of Immunosuppressant (> 300 days)	11,113	74	56,759.46	1.30375	1.508 (0.473,4.809)	0.488	1.857 (0.581,5.937)	0.2967
Hypertension Yes	8135	40	38,322.42	1.04378	0.648 (0.414,1.013)	0.057	1.213 (0.72,2.043)	0.4685
Diabetes mellitus Yes	6251	48	29,495.66	1.62736	1.79 (1.129,2.838)	0.013	1.296 (0.814,2.063)	0.2747
Dyslipidemia Yes	4085	10	18,540.27	0.53937	0.346 (0.178,0.673)	0.002	0.453 (0.19,1.079)	0.0739
Transplanted organ								
1. Kidney	7026	21	33,615.34	0.62471	1 (Ref.)	0.0001	1 (Ref.)	0.3227
2. Liver	5445	54	25,616.58	2.10801	3.398 (2.053,5.626)		2.016 (1.067,3.809)	
3. Pancreas	28	0	105.38	0	N.A		N.A	
4. Heart	398	2	1760.07	1.13632	1.85 (0.434,7.889)		1.288 (0.297,5.592)	
5. Lung	73	0	233.99	0	N.A		N.A	

*NA* not available

Further sub-analyses to determine the risk factor of PC prevalence were performed after adjustment for significantly different variables between the TPL and non-TPL groups, such as age, aspirin use, statin use, hypertension, diabetes, dyslipidemia, and immunosuppressant use (Table 4). Crude model 1, without adjustment of the aforementioned variables, showed that the TPL group had a significantly unfavorable HR for PC prevalence (HR 2.178, CI 1.628–2.912, *p* < 0.001).

**Table 4:** Subgroup analysis adjusted by the risk factors of prostate cancer prevalence

Characteristics	N	Event	Duration	IR (per 1000)	Model 1		Model 2		Model 3	
					HR	*p* value	HR	*p* value	HR	*p* value
Transplantation										
No transplantation	38,910	111	190,764.2	0.58187	1 (Ref.)	< .001	1 (Ref.)	< 0.001	1 (Ref.)	0.1948
Transplantation	12,970	77	61,331.36	1.25548	2.178 (1.628,2.912)	1.965 (1.392,2.772)	0.539 (0.212,1.372)			
Transplanted organ										
Control	38,910	111	190,764.2	0.58187	1 (Ref.)	< .001	1 (Ref.)	< 0.001	1 (Ref.)	0.0229
1. Kidney	7026	21	33,615.34	0.62471	1.08 (0.677,1.722)	1.066 (0.629,1.806)	0.28 (0.102,0.767)			
2. Liver	5445	54	25,616.58	2.10801	3.661 (2.645,5.068)	2.716 (1.876,3.931)	0.729 (0.29,1.832)			
3. Pancreas	28	0	105.38	0	N.A		N.A		N.A	
4. Heart	398	2	1760.07	1.13632	2.013 (0.497,8.151)	1.241 (0.297,5.179)	0.327 (0.062,1.73)			
5. Lung	73	0	233.99	0	N.A		N.A		N.A	

Model 1 Rude

Model 2 Adjusted by the “Age, Aspirin, Statin, Hypertension, Diabetes, Dyslipidemia”

Model 3 Adjusted by the “Age, Aspirin, Statin, Hypertension, Diabetes, Dyslipidemia, Immunosuppressant”

After adjustment for age, aspirin use, statin use, hypertension, diabetes, and dyslipidemia, model 1 showed that the TPL group also had an unfavorable HR, similar to that in crude model 1 (HR 1.965, CI 1.392–2.772). Both models showed that organ TPL subtypes had unfavorable HRs for PC prevalence, for example, HR 3.661 (CI 2.645–5.068) in crude model 1 and HR 2.716 (CI 1.876–3.931) in model 2 (*p* < 0.001) for the liver. Meanwhile, on adjustment for additional immunosuppressant use along with other variables in model 3, PC prevalence (*p* = 0.195) became insignificant in the TPL group and remained significant only in the kidney TPL subtype group (HR 0.28, CI 0.102–0.767).

Multivariate analysis of the overall patient population showed that immunosuppressant use was a significant unfavorable factor for PC prevalence (HR 1.691, CI 1.144–2.498) and that the liver TPL subtype was a significant factor (HR 1.866, CI 1.188–2.93, *p* < 0.001) (Additional file 1: Table S1). The characteristics of each organ TPL group among TPL patients are compared in Additional file 1: Table S2.

**Fig. 1:**
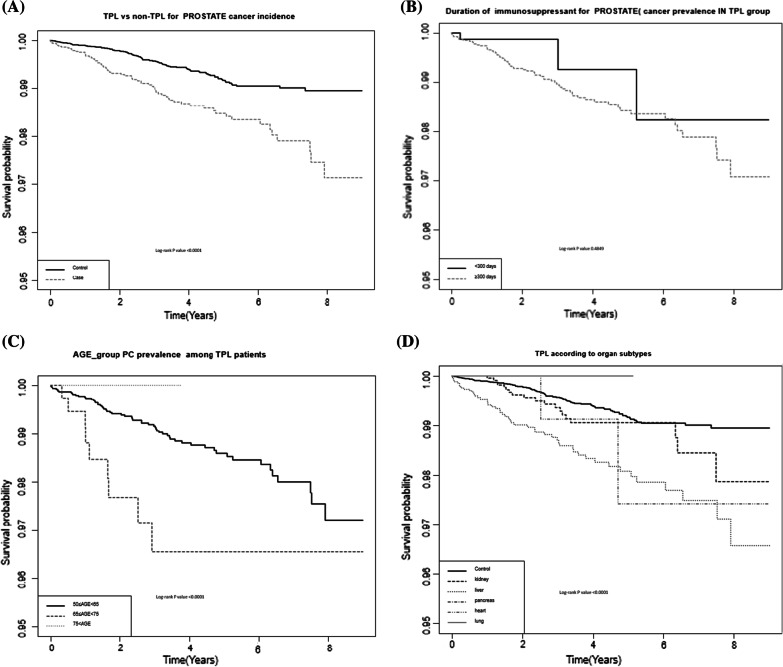
Kaplan–Meier OS curves compared using log-rank test between **a** the TPL and the non-TPL groups; **b** according to the duration of immunosuppressant use, **c** to age group, and **d** the TPL and the non-TPL

Kaplan–Meier analysis of PC incidence showed that the TPL group had a significantly higher PC prevalence than did the non-TPL group (*p* < 0.001, Fig. 1a). Meanwhile, the duration of immunosuppressant use (< 300 days or > 300 days) did not significantly affect the difference in PC prevalence among the TPL groups (*p* = 0.4849, Fig. 1b), but the age was significantly different according to the age subgroups (*p* < 0.001, Fig. 1c). The organ subtype curves also significantly differentiated PC prevalence (*p* < 0.001, Fig. 1d).

## Discussion

Newly diagnosed PC after TPL has become an important issue of TPL because of the prolonged lifespan by the resolution of the end-staged organ, introduction of prostate-specific antigen (PSA) screening for early detection of PC, and concerns about the possibility of aggressive PC behavior with rapid cancer progression and poor survival prognoses in the setting of immunosuppression [6, 8–10]. This population-based study matched by age and year of TPL surgery showed a significantly independent relation of PC prevalence with TPL and organ subtype and with immunosuppressant use and its duration (Tables 2 and 4). Among TPL patients, age was the only significant factor in terms of PC prevalence (Table 3). This implied that routine age-dependent PC screening programs in the general population should also be applied to recipient patients after TPL [11].

Previous studies published conflicting results about the relation between TPL and PC incidence [11–13]. Some researchers reported an increased risk of PC [12], with an estimated cumulative incidence rate of 0.72% and 1.74%, 1 and 3 years after transplantation, respectively [13], and other researchers denied significant associations between TPL and PC incidence [4, 8, 14–16]. They explained that immunosuppression did not affect the natural history of newly diagnosed PC because most new PCs were clinically-insignificant and low-risk and the major cause of death in these cases was primary organ failure or transplant organ failure [14, 15] resulting in a lower life expectancy that that of TPL recipients [9, 17, 18]. These conflicting conclusions were affected by differences in cohort characteristics, immunosuppressant duration, and institutional therapeutic measures and thus no definite conclusion can be drawn until a randomized clinical trial is carried out.

Immunosuppression regimens and the duration of these regimens may act as a double-edged sword with regard to increased graft survival, as they can potentially increase the risk of malignancy, particularly in consideration of the longer life expectancy. Similar to previous studies [19, 20], this study showed that PC incidence was significantly influenced by both TPL and immunosuppressant use (> 300 days). Considering the change in HRs for TPL with immunosuppressant use, the effect of immunosuppressant use was considered a more prominent contributor to increased PC incidence than TPL itself, as well as other well-known risk factors such as age. This is because the effect, as shown by the HR, changed from a favorable effect (HR < 1.0) among the overall cohort to an unfavorable effect (HR > 1.0) with TPL cohort only, on PC incidence. This change was due to the additional adjustment of the use of immunosuppressants in the multivariate analysis (models 3 and 4 in Table 4 and Additional file 1: Table S1) after adjusting for the well-known risk factors of PC incidence. As almost all the TPL patients had used immunosuppressants (99.9%) for longer than 300 days (85.7%), the effect of multicollinearity between immunosuppressant use and TPL affected the instability of the HR for TPL in PC incidence (*p* > 0.05, Table 3). Additionally, to further estimate the significant effect of immunosuppressant use on PC incidence, models 2 and 3 in Table 4 show that well-known risk factors such as age, which was also a significantly independent factor in this study (*p* < 0.001, Table 2), did not have an effect on the HR of PC incidence. However, the immunosuppressant use changed the effect of TPL and its organ subtypes on PC incidence, as shown by the HR. This means that the use of immunosuppressants was strongly influenced by the PC incidence as well as by the existence of multicollinearity between the immunosuppressant use and the TPL.

Immunosuppressants such as calcineurin inhibitors (cyclosporine and tacrolimus), sirolimus, and azathioprine have been known to increase the risk of cancer by inhibiting the protective effects of the immune system against carcinogenesis [21, 22]. However, a recent meta-analytic study showed that the association between sirolimus use and a higher PC incidence (incidence rate ratio: 1.06 and 1.85, respectively) was influenced by the higher risk of clinically-insignificant low-risk PC rather than by high-risk PC and by the effect of detection bias and screening effects rather than sirolimus carcinogenesis itself [22]. In addition, mTOR inhibitors have replaced cycloserine and tacrolimus because of their recently reported anti-tumor effects because of which they have a lower risk for cancer than that associated with previous immunosuppressant agents [23]. Collectively, it would be more convenient to establish active PC screening, monitoring, and therapeutic guidelines for high-risk PC rather than focusing on the increase in low-risk PC after TPL, proportional to the longer duration of immunosuppressant.

A subgroup analysis was carried out according to the TPL organ type in this study, and liver and kidney TPL patients were found to have a significant risk for PC. In the unadjusted multivariate analyses, kidney TPL (HR 1.08/1.066 in models 1 and 2) and liver TPL (HR 3.661/2.716 in models 1 and 2) were found to be significantly associated with an increase in the incidence of PC (Table 4). However, on adjustment of immunosuppressant use, only kidney TPL was found to be significantly associated with PC (HR 0.28, *p* = 0.0229). Patients with liver TPL had a higher PC incidence, particularly older patients (51.29 ± 8 years old); they also had a poorer OS than that in other organ TPL groups (Additional file 1: Table S2). The worse survival outcomes in the liver TPL group are because of the risk of liver failure after TPL, with a higher incidence of comorbidities such as liver cirrhosis and hepatitis [24], whereas kidney TPL recipients are free from end-stage renal disease and dialysis-related mortality, resulting in better survival outcomes than for other TPL subtypes [17, 21].

Many TPL-related associations provided guidelines on the timing and indication of PC screening before/after TPL and on therapeutic measures for PC [24–26]. They suggested initial screening at age 50 years with a life expectancy greater than 10 years, and that high-risk localized and metastatic PC should be treated. Furthermore, the PC-high risk populations for early prevalence of PC with aggressive prognostic feature are high priorities for the PC screening and treatment, such as African American, and BRCA-positive family history [27, 28]. They recommended that overuse of PC screening and active treatment for low-risk localized PC (such as < PSA ng/dL) be avoided so that TPL surgery can be performed without wasting time waiting for 2–5 years to attain a cancer-free status, considering most patients (about 70%) on hemodialysis die within 5 years [29]. However, because of the lack of randomized trials, it was unclear whether PC screening should be a part of the routine workup for TPL eligibility and post-TPL surveillance until a recent meta-analytic study on PC screening in TPL patients suggested the necessity for PC guidance.

This study has some inherent limitations relating to its retrospective design, the small number of lung and pancreas TPL patients, and the absence of pathological data (i.e., TNM stage and pathologic grade) and laboratory parameters such as the prostate-specific antigen (PSA) information. This information could differentiate between the clinically significant and insignificant PC incidence considering the important clinical indication for PC therapy that can currently help to analyze the immunosuppressants effect on an under/over-estimated PSA level. In addition, the PSA information might also adjust the effect of PSA screening bias because the TPL group had a higher chance of undergoing PSA examination due to their highly frequent and thorough hospital examination. Body mass index, underlying disease, and medications that have synergistic effects with each immunosuppressants were not evaluated either. It is the cancer specific survival data that are important in this study because we could not confirm survival on either TPL or PC. Further study should be considered after adjustment of all these potential variables affecting on the incidence of prostate cancer in TPL group to demonstrate the significantly direct association of immunosuppressant use with the prostate cancer incidence. Despite these limitations, to the best of our knowledge, this study is the first Korean TPL-PC study, and it shows the effect of immunosuppressant use and duration on PC incidence.

## Conclusion

The study showed that PC incidence in TPL patients is significantly affected by immunosuppressant use and its duration of use, as well as organ TPL subtypes. As age, as a nonmodifiable PC risk factor, can affect the cancer mortality rate in this immunosuppressed population, PC screening should be considered in liaison with the patients’ age; active treatment is also recommended in TPL patients who are at a high risk of developing PC. Thus, further longitudinal studies with PSA levels should be designed to evaluate clinically significant PC incidence according to the TPL organ subtype and the duration of immunosuppressants used.

## Supplementary Information

**Additional file 1.** The comparison of characteristics between TPL group among TPL patients. **Supplementary Table 1.** Univariate & Multivariate cox regression for prostate cancer incidence among overall patients including the immunosuppressant. **Supplementary Table 2.** Comparison of baseline characteristics according to the transplanted organ.

## Data Availability

The data that support the findings of this study are available from the IRB of National Cancer Center, Goyang, Korea (e-mail, eirb@ncc.re.kr). Data are available from the authors upon available requests and with permission of the IRB of National Cancer Center.

## Abbreviations

PC: Prostate cancer
TPL: Organ-transplantation
HTN: Hypertension
DM: Diabetes mellitus
NHIS: The Korean National Health Insurance System
ICD-10-CM: The international classification of diseases, tenth revision, clinical modification
PSA: Prostate-specific antigen
HR: Hazard ratio
CI: 95% confidence intervals
