# Supplementary material for: The effect of subsequent immunosuppressant use in organ-transplanted patients on prostate cancer incidence: a retrospective analysis using the Korean National Health Insurance Database
Source: BMC Urol. 2021 Aug 21;21:112. doi: 10.1186/s12894-021-00883-8 (PMC8379856; doi:10.1186/s12894-021-00883-8)

Supplementary Table 1. Univariate & Multivariate cox regression for prostate cancer incidence among overall patients including the immunosuppressant

|  |  |  |  |  | Univariate | | Multivariate | |
| --- | --- | --- | --- | --- | --- | --- | --- | --- |
| Parameters | N | event | Duration | IR(per 1000) | HR | P-value | HR | P-value |
| Demographics |  |  |  |  |  |  |  |  |
| Age (in years) group |  |  |  |  |  | <.0001 |  | <.0001 |
| 20≤Age<50 | 25856 | 18 | 135209.95 | 0.1331 | 0.098(0.06,0.161) |  | 0.115(0.07,0.189) |  |
| 50≤Age<65 | 24308 | 144 | 110258.86 | 1.306 | 1(Ref.) |  | 1(Ref.) |  |
| 65≤Age≤75 | 1688 | 25 | 6550.21 | 3.8167 | 3.089(2.019,4.726) |  | 2.979(1.938,4.579) |  |
| 75< | 28 | 1 | 76.54 | 13.0646 | 12.92(1.803,92.597) |  | 13.509(1.872,97.471) |  |
| Medication |  |  |  |  |  |  |  |  |
| Aspirin |  |  |  |  |  |  |  |  |
| No | 50104 | 175 | 244077.7 | 0.71698 | 1(Ref.) | 0.0038 | 1(Ref.) | 0.4193 |
| Yes | 1776 | 13 | 8017.85 | 1.62138 | 2.3(1.309,4.04) |  | 1.272(0.709,2.283) |  |
| Statin |  |  |  |  |  |  |  |  |
| No | 43179 | 151 | 213020.46 | 0.70885 | 1(Ref.) | 0.0905 | 1(Ref.) | 0.3365 |
| Yes | 8701 | 37 | 39075.1 | 0.94689 | 1.364(0.952,1.955) |  | 1.299(0.762,2.212) |  |
| Immunosuppressant |  |  |  |  |  |  |  |  |
| no | 30302 | 72 | 148929.9 | 0.48345 | 1(Ref.) | <.0001 | 1(Ref.) | 0.0084 |
| yes | 21578 | 116 | 103165.66 | 1.12441 | 2.336(1.741,3.135) |  | 1.691(1.144,2.498) |  |
| Hypertension |  |  |  |  |  |  |  |  |
| No | 36369 | 108 | 180044.64 | 0.59985 | 1(Ref.) | <.0001 | 1(Ref.) | 0.0742 |
| Yes | 15511 | 80 | 72050.92 | 1.11033 | 1.878(1.407,2.508) |  | 1.348(0.971,1.87) |  |
| Diabetes mellitus |  |  |  |  |  |  |  |  |
| No | 42376 | 124 | 207980.85 | 0.59621 | 1(Ref.) | <.0001 | 1(Ref.) | 0.2973 |
| Yes | 9504 | 64 | 44114.7 | 1.45076 | 2.461(1.82,3.328) |  | 1.209(0.846,1.727) |  |
| Dyslipidemia |  |  |  |  |  |  |  |  |
| no | 43334 | 159 | 214609.86 | 0.74088 | 1(Ref.) | 0.7258 | 1(Ref.) | 0.1146 |
| yes | 8546 | 29 | 37485.7 | 0.77363 | 1.073(0.722,1.595) |  | 0.627(0.351,1.12) |  |
| Transplanted organ |  |  |  |  |  |  |  |  |
| Control | 38910 | 111 | 190764.2 | 0.58187 | 1(Ref.) | <.0001 | 1(Ref.) | 0.0179 |
| 1.kidney | 7026 | 21 | 33615.34 | 0.62471 | 1.08(0.677,1.722) |  | 0.74(0.413,1.326) |  |
| 2.liver | 5445 | 54 | 25616.58 | 2.10801 | 3.661(2.645,5.068) |  | 1.866(1.188,2.93) |  |
| 3.pancreas | 28 | 0 | 105.38 | 0 | . |  | . |  |
| 4.heart | 398 | 2 | 1760.07 | 1.13632 | 2.013(0.497,8.151) |  | 0.864(0.203,3.683) |  |
| 5.lung | 73 | 0 | 233.99 | 0 | . |  | . |  |

Supplementary Table 2. Comparison of baseline characteristics according to the transplanted organ

| Parameter | Control | Kidney | Liver | Pancreas | Heart | Lung |  |
| --- | --- | --- | --- | --- | --- | --- | --- |
| n | 38910 | 7026 | 5445 | 28 | 398 | 73 |  |
| Age (years; mean, STD) | 48.1±10.24 | 45.6±10.88 | 51.29±8 | 35.25±11.48 | 49.31±12.25 | 49.93±12.47 | <.0001 |
| Age group |  |  |  |  |  |  | <.0001 |
| >20-49 | 19392 (49.84) | 4228 (60.18) | 2003 (36.79) | 24 (85.71) | 183 (45.98) | 26 (35.62) |  |
| >50-64 | 18231(46.85) | 2609 (37.13) | 3243 (59.56) | 3 (10.71) | 177 (44.47) | 45 (61.64) |  |
| >65-74 | 1266 (3.25) | 186 (2.65) | 197 (3.62) | 1 (3.57) | 37 (9.3) | 1 (1.37) |  |
| ≥75 | 21 (0.05) | 3 (0.04) | 2 (0.04) | 0 (0) | 1 (0.25) | 1 (1.37) |  |
| Year of prostate cancer diagnosis | |  |  |  |  |  | 0.1036 |
| 2007-2009 | 0 (0) | 1 (4.76) | 0 (0) | 0 (0) | 0(0) | 0(0) |  |
| 2010-2012 | 20 (18.02) | 5 (23.81) | 14 (25.93) | 0 (0) | 1 (50) | 0 (0) |  |
| 2013-2015 | 91 (81.98) | 15 (71.43) | 40 (74.07) | 0 (0) | 1 (50) | 0 (0) |  |
| Year of transplantation |  |  |  |  |  |  | <.0001 |
| 2003-2007 | 9393 (24.14) | 1592 (22.66) | 1431 (26.28) | 4 (14.29) | 101 (25.38) | 3 (4.11) |  |
| 2008-2012 | 13899 (35.72) | 2525 (35.94) | 1957 (35.94) | 5 (17.86) | 124(31.16) | 22 (30.14) |  |
| 2013-2015 | 15618 (40.14) | 2909 (41.4) | 2057 (37.78) | 19 (67.86) | 173 (43.47) | 48 (65.75) |  |
| Underlying disease |  |  |  |  |  |  |  |
| Hypertension | 7376 (18.96) | 6256 (89.04) | 1533 (28.15) | 7 (25) | 317 (79.65) | 22 (30.14) | <.0001 |
| Diabetes | 3253 (8.36) | 3059 (43.54) | 2941 (54.01) | 12 (42.86) | 209 (52.51) | 30 (41.1) | <.0001 |
| Dyslipidemia | 4461 (11.46) | 3386 (48.19) | 435 (7.99) | 4 (14.29) | 244 (61.31) | 16 (21.92) | <.0001 |
| Medication |  |  |  |  |  |  |  |
| Aspirin | 803 (2.06) | 569 (8.1) | 342 (6.28) | 2 (7.14) | 50 (12.56) | 10 (13.7) |  |
| Statin | 4389 (11.28) | 3417 (48.63) | 555 (10.19) | 3 (10.71) | 318 (79.9) | 19 (26.03) |  |
| Charlson Comorbidity Index grade 2 | |  |  |  |  |  | <.0001 |
| 0 | 23689 (60.88) | 1 (0.01) | 2 (0.04) | 0 (0) | 1 (0.25) | 0 (0) |  |
| 1 | 8126 (20.88) | 1 (0.01) | 81 (1.49) | 2 (7.14) | 15 (3.77) | 6 (8.22) |  |
| 2 or more | 7095 (18.23) | 7024 (99.97) | 5362 (98.48) | 26 (92.86) | 382 (95.98) | 67 (91.78) |  |
| Total |  |  |  |  |  |  |  |
| Survival time (year) | 4.9±2.49 | 4.78±2.46 | 4.7±2.53 | 3.76±2.56 | 4.42±2.54 | 3.21±1.65 | <.0001 |
| Duration of immunosuppressant use (≥300 days) | 225 (0.58) | 6152 (87.56) | 4550 (83.56) | 18 (64.29) | 342 (85.93) | 51 (69.86) | <.0001 |


Supplement: Supplementary file 1 — Additional file 1. The comparison of characteristics between TPL group among TPL patients. Supplementary Table 1. Univariate & Multivariate cox regression for prostate cancer incidence among overall patients including the immunosuppressant. Supplementary Table 2. Comparison of baseline characteristics according to the transplanted organ. [file 12894_2021_883_MOESM1_ESM.docx]
